# Global loss of a nuclear lamina component, lamin A/C, and LINC complex components SUN1, SUN2, and nesprin-2 in breast cancer

**DOI:** 10.1002/cam4.495

**Published:** 2015-07-14

**Authors:** Ayaka Matsumoto, Miki Hieda, Yuhki Yokoyama, Yu Nishioka, Katsuhide Yoshidome, Masahiko Tsujimoto, Nariaki Matsuura

**Affiliations:** 1 Graduate School of Medicine and Health Science, Osaka UniversitySuita, Japan; 2 Present Institution, Carna Bioscience, Inc.Kobe, Japan; 3 Department of Breast Surgery, Osaka Police HospitalOsaka, Japan; 4 Department of Pathology, Osaka Police HospitalOsaka, Japan

**Keywords:** Breast cancer, lamin A/C, LINC complex, nesprin-2, SUN1/2

## Abstract

Cancer cells exhibit a variety of features indicative of atypical nuclei. However, the molecular mechanisms underlying these phenomena remain to be elucidated. The linker of nucleoskeleton and cytoskeleton (LINC) complex, a nuclear envelope protein complex consisting mainly of the SUN and nesprin proteins, connects nuclear lamina and cytoskeletal filaments and helps to regulate the size and shape of the nucleus. Using immunohistology, we found that a nuclear lamina component, lamin A/C and all of the investigated LINC complex components, SUN1, SUN2, and nesprin-2, were downregulated in human breast cancer tissues. In the majority of cases, we observed lower expression levels of these analytes in samples' cancerous regions as compared to their cancer-associated noncancerous regions (in cancerous regions, percentage of tissue samples exhibiting low protein expression: lamin A/C, 85% [*n* = 73]; SUN1, 88% [*n* = 43]; SUN2, 74% [*n* = 43]; and nesprin-2, 79% [*n* = 53]). Statistical analysis showed that the frequencies of recurrence and HER2 expression were negatively correlated with lamin A/C expression (*P *<* *0.05), and intrinsic subtype and ki-67 level were associated with nesprin-2 expression (*P *<* *0.05). In addition, combinatorial analysis using the above four parameters showed that all patients exhibited reduced expression of at least one of four components despite the tumor's pathological classification. Furthermore, several cultured breast cancer cell lines expressed less SUN1, SUN2, nesprin-2 mRNA, and lamin A/C compared to noncancerous mammary gland cells. Together, these results suggest that the strongly reduced expression of LINC complex and nuclear lamina components may play fundamental pathological functions in breast cancer progression.

## Introduction

The nuclear architecture provides a framework for organizing and regulating the diverse functional processes within cells. There are significant differences in nuclear architecture between cancer and normal cells 1,2. Nuclear deformations are used in pathological diagnosis and some anticancer treatments restore normal nuclear structure and function. However, features of nuclear organization and their functional significance have been poorly understood.

The nucleus is surrounded by a double lipid bilayer, the nuclear envelope, comprising the inner and outer nuclear membrane (INM and ONM). The LINC (linker of nucleoskeleton and cytoskeleton) complex, a multifunctional NE protein assembly that maintains nuclear structure, is composed primarily of two kinds of protein families: nesprin proteins, which are recruited to the outer nuclear membrane, where they interact with the cytoskeleton; SUN proteins, located in the inner nuclear membrane, where they interact with lamins in the nucleoplasm and with nesprin proteins in the perinuclear space 3,4. Lamin proteins, which are intermediate filaments located under the nuclear envelope, are components of nuclear lamina that function as a nuclear skeleton. Each of these proteins is composed of multiple variants derived from different genes and alternative splicing. The LINC complex plays roles in many cellular processes, and is involved not only in controlling nuclear size, shape, and structure, but also in cell migration, polarization, and differentiation 5. Abnormalities of LINC complex and nuclear lamina proteins are associated with many human diseases. For instance, lamin A/C gene mutants result in laminopathies that include Emery-Dreifuss muscular dystrophy (AD-EDMD); Hutchinson-Gilford progeria, a premature aging syndrome (HGPS); and dilated cardiomyopathy 6. Deregulated lamin expression has been observed in various cancers. Loss of lamin A/C expression has been reported in colon cancer 7, small cell lung cancer 8, leukemias and lymphomas 9,10, ovarian cancer 11, and breast cancer 12,13. On the other hand, overexpression of lamin A/C has been reported in ovarian cancers 14, colorectal cancer 15, prostate cancer 16, and skin cancer 17,18. Lamin A/C is directly associated with chromatin 19,20, and is therefore believed to confer nuclear rigidity and chromatin stability. Perinuclear chromosome tethering is essential for the maintenance of genome integrity 21. Recent studies showed that SUN1 plays a role in telomere tethering during postmitotic nuclear assembly 22. However, the neoplastic regulation and protein levels of the LINC complex components, that are, SUN and nesprin proteins, and the clinical significance of the LINC complex in different cancer types, still have not yet been well elucidated. Hence, in this study we examined the protein expression levels of a nuclear lamina component, lamin A/C, and three LINC complex components, SUN1, SUN2, and nesprin-2 in breast cancer tissue.

## Materials and Methods

### Immunohistochemical staining

Samples were obtained from patients with breast cancer who underwent surgery between 2000 and 2004 at Osaka Police Hospital (Osaka, Japan). The samples were acquired and immunostained with the informed consent of patients and the approval of the Osaka Police Hospital Ethics Committee according to the institutional Ethical and Legal rules. The detailed clinical subtypes of the patients are enumerated in Table1. Formalin-fixed, paraffin-embedded specimens were stained as described previously 23,24. Antigen retrieval was performed in 0.01 mol/L citrate buffer (pH 6.0) or DAKO Target Retrieval solution (pH 9.0) using a Pascal pressure chamber (DAKO, Produktionsvej, Denmark). Goat anti-lamin A/C (sc-6215; Santa Cruz Biotechnology, Dallas, TA, USA), rabbit anti-SUN1 (HPA008346; Sigma–Aldrich, St. Louis, MO, USA), rabbit anti-SUN2 (06-1038; Millipore, Billerica, MA, USA), and rabbit anti-nesprin-2 pAbs (HPA003435; Sigma-Aldrich, St. Louis, MO, USA) were used as primary antibodies. Specificities of the antibodies were validated in Fig. S1. All primary antibodies were diluted as 1:200. Proteins were detected using biotinylated anti-goat IgG antibody (DAKO, Denmark) and peroxidase-conjugated streptavidin (DAKO, Denmark) for lamin A/C, and Histofine Simple Stain MAX PO (MULTI, Nichirei Bioscience Inc., Tokyo, Japan) for SUN1, SUN2 and nesprin-2. Samples were visualized with 3,3-diaminobenzidine (DAB) solution (Sigma-Aldrich, USA) and counterstained with hematoxylin. The immunohistochemical staining of tissues was assessed by at least two pathologists.

**Table 1:** Clinical subtypes of patients

	Used for SUN1 analysis	Used for SUN2 analysis	Used for lamin A/C analysis	Used for nesprin-2 analysis
Luminal A	19	19	35	22
Luminal B	5	5	10	8
HER2^+^	5	5	9	7
Triple negative	12	12	17	14
Unknown	2	2	2	2
Total	43	43	73	53

### Statistical analysis

All results were presented as mean ± SD. For clinicopathological analysis, the Chi-squared test was applied. Univariate survival analyses were carried out using Kaplan–Meier analysis and the log-rank test. *P* values <0.05 were considered statistically significant.

### Cell culture

The maintenance of human breast cancer cells MDA-MB-231, MCF7, ZR75-30, and human mammary epithelial cells MCF10A (American Type Culture Collection) have been described 25. Human cervix adenocarcinoma HeLa cells were grown in DMEM (Wako Pure Chemical, Osaka, Japan) supplemented with 10% (w/v) FBS.

### Real-time PCR

Total RNA of cultured cells was isolated using the PureLink RNA Mini Kit (Life technology, Carlsbad, CA) and reverse transcribed using Prime Script RT reagent (Takara, Shiga, Japan). Total RNA from human mammary epithelial and human breast cancer tissues was purchased from Takara. The mRNA levels of *SUN1, SUN2, nesprin-2,* and *lamin A/C* were quantitated by real-time PCR using LightCycler SYBR Green I Master and a LightCycler 480 System (Roche Diagnostics, Basel, Switzerland), and normalized to the mRNA level of *GAPDH* (encoding glyceraldehyde-3-phosphate dehydrogenase). Sequences of the primers used are as follows: for *SUN1*, 5'-AAGTCAGAGAAATGGTGAAACTCC-3' and 5'-TCACAAACTGTGATGAGAACCTCT-3'; for *SUN2*, 5'-CTTCCTCGGGCTACTCCTCT-3' and 5'-GCCACCATCCAGAGTAAGGA-3'; for GAPDH, 5'-AATCCCATCACCATCTTCC-3' and 5'-GCAGAGATGATGACCCTTT-3'; for *nesprin-2*, 5'-GGAGAAAGTTGGTTTCAAAAACTC-3' and 5'-AAAGTGGGCTGATCCTGTTTT-3'; and for *lamin A/C*, 5'-AGCAAAGTGCGTGAGGAGTT-3' and 5'-AGGTCACCCTCCTTCTTGGT-3'.

## Results

### Immunohistochemistry of LINC complex components

**Figure 1:**
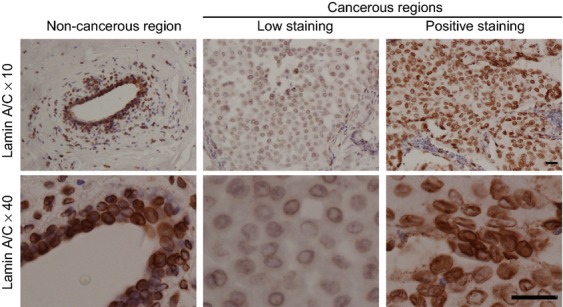
Immunohistochemical staining of lamin A/C. Specimens of breast tumor were stained using anti-lamin A/C pAbs. Representative cases including noncancerous (left) and cancerous regions (middle and right) are shown. The upper panels were obtained at lower magnification than the lower panels. Bar, 50 *μ*m.

To examine the expression levels of a nuclear lamina component, lamin A/C and three LINC complex components in breast cancer tissues, immunohistochemistry was performed using anti-lamin A/C, anti-SUN1, anti-SUN2, and anti-nesprin-2 specific pAbs. The performance of these antibodies has been validated in western blotting and immunofluorescence microscopy (Fig. S1). The detailed clinical subtypes of the patients are enumerated in Table1. Anti-lamin A/C pAbs stained cancer-associated noncancerous mammary epithelial and myoepithelial cells at the nuclear membrane (Fig.1, left column). Consistent with a previous result 13, most cases showed that lamin A/C staining was less intense in cancerous regions (Fig.1, middle column) than in cancer-associated noncancerous regions (Fig.1, left column), although some cases showed normal expression (Fig.1, right column). Normal mammary epithelial cells form intact duct structure surrounded by myoepithelial cells. This structure is disrupted in cancer. Thus intact duct structure in adjacent cancer region can be defined as cancer-associated noncancerous mammary epithelial cells.

**Figure 2:**
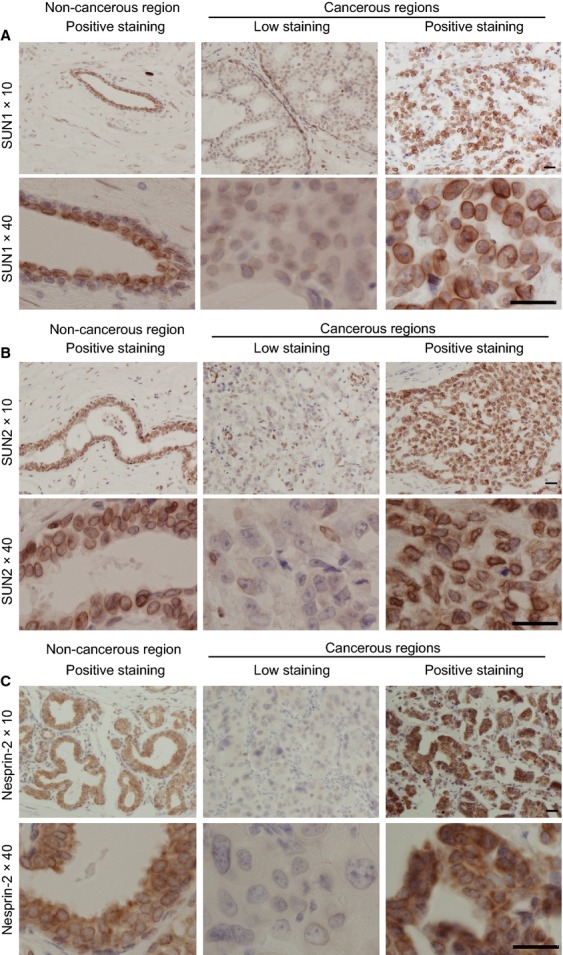
Immunohistochemical staining for SUN1, SUN2, and nesprin-2. Specimens of breast tumor were stained using pAbs against SUN1 (A), SUN2 (B), and nesprin-2 (C). Representative cases including noncancerous (left) and cancerous regions (middle and right) are shown. The upper panels were obtained at lower magnification than the lower panels. Bar, 50 *μ*m.

In addition, all cancer-associated noncancerous mammary epithelial and myoepithelial cells were stained with anti-SUN1, anti-SUN2, and anti-nesprin-2 pAbs at the nuclear membrane (Fig.2 left column). In many cases, the staining intensities of SUN1, SUN2, and nesprin-2 were often weaker in cancer cells (Fig.2, middle column) than in noncancerous regions (Fig.2, left column); again, however, some cases exhibited normal expression (Fig.2, right column).

### Expression of LINC complex and nuclear lamina components is robustly reduced in breast tumor regions

**Figure 3:**
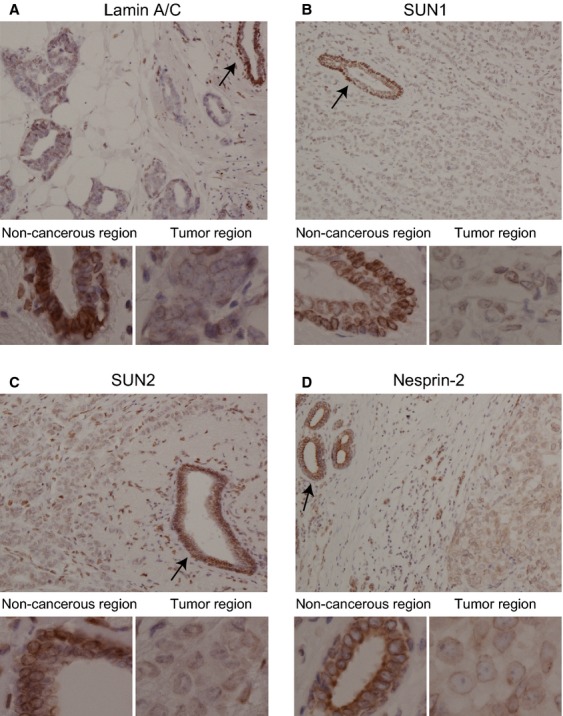
Loss of lamin A/C, SUN1, SUN2, and nesprin-2 staining in the cancerous regions of breast cancer tissue. (A–D) Noncancerous and cancerous regions of breast cancer tissue were stained with anti-lamin A/C (A), anti-SUN1 (B), anti-SUN2 (C), and anti-nesprin-2 (D) pAbs; representative staining patterns are shown. The upper panels were obtained at lower magnification than the lower panels. Most myoepithelial cells stained more strongly for anti-SUN1 pAbs in cancer-associated noncancerous regions than in most mammary epithelial cells, but not in all cases (see Fig.2).

**Figure 4:**
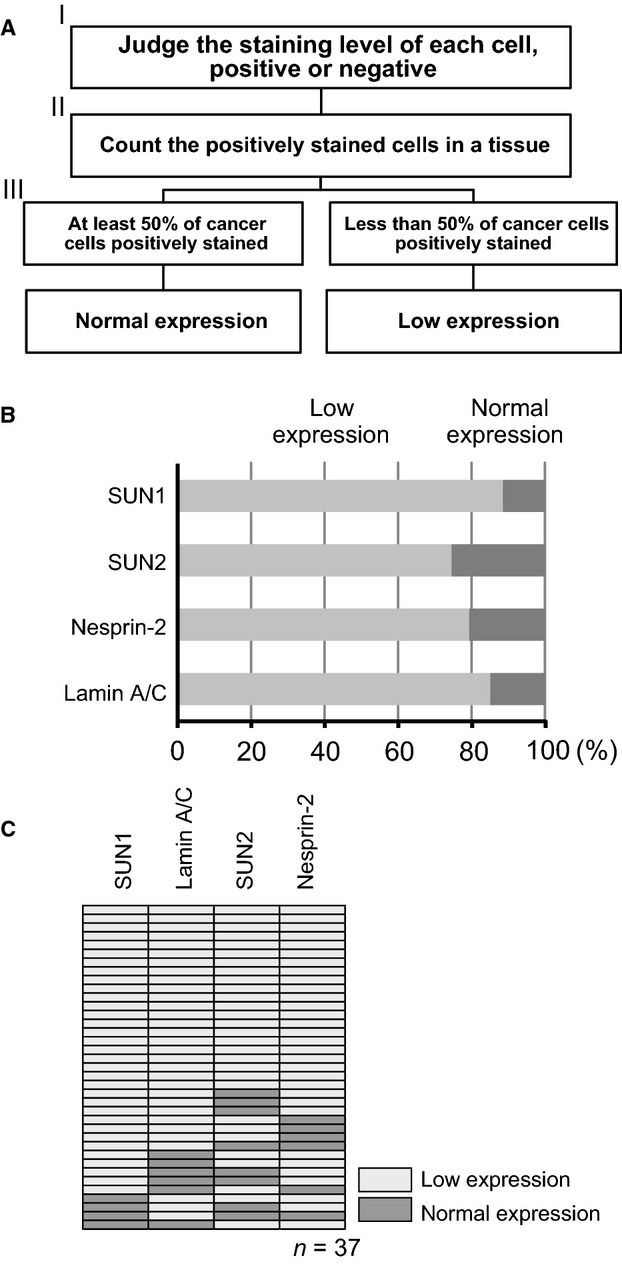
Quantitation of staining of LINC complex and nuclear lamina components. (A) A method for quantitation of LINC complex and nuclear lamina protein expression in cancerous regions. The staining pattern of each protein in tumor tissue was used to categorize cells into normal and low expression groups. Staining of mammary epithelial cells in a cancer-associated noncancerous region in the same tissue was used as an internal positive control. The staining patterns were classified into normal and low expression based on the percentage of positively stained cancer cells in the tissue. (B) Bars indicate the percentages of tissues in the low expression and normal expression groups for the indicated antibodies. (C) This panel indicates the expression of each protein. Each horizontal line represents the expression in one patient. Dark gray and light gray boxes indicate normal and low expression, respectively. LINC, linker of nucleoskeleton and cytoskeleton.

Anti-lamin A/C, anti-SUN1, anti-SUN2, and anti-nesprin-2 pAbs clearly stained noncancerous acini of terminal-duct lobular units adjacent to cancer cells at the nuclear membrane (Fig.3A–D, arrows in upper panels). We often observed lower staining of these LINC complex and nuclear lamina components, even within a single breast cancer tissue sample (Fig.3A–D, upper panels). The lower panels in Fig.3A–D show the staining in noncancerous and tumor regions at higher magnification. These results showed that expression of all the investigated components were present at lower levels in tumor regions than in noncancerous regions. To quantitate the reduction of these proteins expression in cancerous regions, we divided the immunohistochemically stained tissues into two categories, “low expression” and “normal expression,” based on staining intensity and percentage of stained cancer cells these groups are defined below (Fig.4A). Cancer-associated noncancerous mammary epithelial and myoepithelial cells exhibited strong SUN1, SUN2, nesprin-2, and lamin A/C staining (Fig.1, left column; Fig.2, left column; and Fig.3A–D, arrows in upper panels) and could be used as internal positive controls for staining. Positive staining was defined as either the same or stronger staining intensity in cancer cells compared with noncancerous cells in the same tissue. Negative staining was defined as weaker staining intensity in cancer cells compared with noncancerous cells. Using this definition, we first judged whether each cancer cell was stained (positive) or not (negative) (Fig.4A, I). Next, we calculated the ratio of positively stained cells in a tissue (Fig.4A, II), and classified these tissues into two categories: the low expression group, in which fewer than 50% of cancer cells were positively stained, and the normal expression group, in which at least 50% of cancer cells were positively stained (Fig.4A, III). This quantitation revealed that for all four proteins, most cancer tissues were in the low expression group: 85.1%, 88.4%, 74.4%, and 79.2% for lamin A/C, SUN1, SUN2, and nesprin-2, respectively (Fig.4B).

We then analyzed the combination of the four proteins in each patient (Fig.4C) and identified two important points. First, tissues from more than half of the patients showed low expression of all four LINC complex and nuclear lamina proteins. Second, all patients exhibited reduced expression of at least one of four components; in other words, no patient expressed normal levels of all four proteins.

### Loss of nesprin-2 and lamin A/C expression correlates with clinicopathological status

Since breast cancers consist of heterogeneous populations of cells, we evaluated the correlation between expression of LINC complex proteins and clinicopathological status (Table2 and Table S1). The reduced lamin A/C expression was associated with HER2 expression (*P *< 0.05) and frequency of recurrence (*P *< 0.05). In addition, all cases with either high HER2 expression (*n* = 17) or positive tumor recurrence (*n* = 18) expressed remarkably low levels of lamin A/C. Furthermore, nesprin-2 expression correlated with invasiveness (*P *< 0.05), intrinsic subtype (*P *< 0.05), and MIB-1 index (*P *< 0.05). Therefore, we performed disease-free and overall survival analysis and log-rank test using the data for all patients or the data excluding DCIS patients. These results showed some correlation between lower expression of lamin A/C or nesprin-2 and disease-free survival, whereas we did not observe statistical significance for either lamin A/C or nesprin-2 expression in both analyses (Fig. S2 and data not shown).

**Table 2:** Cinicopathological parameters in patients with breast cancer

	SUN1			SUN2			nesprin-2			lamin A/C		
Group	Low	Normal	*P*	Low	Normal	*P*	Low	Normal	*P*	Low	Normal	*P*
	*n* = 38	*n* = 5		*n* = 32	*n* = 11		*n* = 42	*n* = 11		*n* = 62	*n* = 11	
	88.4%	11.6%		74.4%	25.6%		79.2%	20.8%		84.9%	15.1%	
Nuclear grade	*n* = 42		0.096	*n* = 42		0.987	*n* = 52		0.664	*n* = 72		0.984
Grades 1/2	22	1		17	6		22	6		37	6	
Grade 3	15	4		14	5		20	4		25	4	
Invasiveness	*n* = 43		0.446	*n* = 43		0.218	*n* = 53		**0.011**	*n* = 73		0.548
DCIS/micro inv	4	0		4	0		3	4		9	1	
Others	34	5		28	11		39	7		53	10	
Pathological classification	*n* = 43		0.709	*n* = 43		0.636	*n* = 53		0.074	*n* = 73		0.557
DCIS, micro inv	4	0		4	0		3	4		9	1	
pap	12	1		9	4		15	4		21	2	
sol	4	0		3	1		3	1		6	2	
sci	9	2		9	2		10	0		17	2	
others	9	2		7	4		11	2		9	4	
Alive/Dead	*n* = 37		0.648	*n* = 37		0.482	*n* = 47		0.235	*n* = 65		0.303
Alive	28	4		24	8		33	8		50	9	
Dead	4	1		3	2		6	0		6	0	
ER	*n* = 42		0.891	*n* = 42		0.612	*n* = 52		0.112	*n* = 72		0.698
Negative	16	2		14	4		20	2		26	4	
Positive	21	3		17	7		22	8		35	7	
PgR	*n* = 41		0.762	*n* = 41		0.524	*n* = 51		0.618	*n* = 70		0.845
Negative	17	2		13	6		20	4		28	5	
Positive	19	3		17	5		21	6		32	5	
HER2	*n* = 43		0.855	*n* = 43		0.644	*n* = 53		0.108	*n* = 73		**0.047**
0/1+	29	4		24	9		33	6		45	11	
2+/3+	9	1		8	2		9	5		17	0	
Intrinsic subtype	*n* = 41		0.711	*n* = 41		0.476	*n* = 51		**0.037**	*n* = 71		0.560
LA	16	3		13	6		18	4		29	6	
LB	5	0		4	1		4	4		9	1	
HER2-like	4	1		4	1		5	2		9	0	
TN	11	1		9	3		14	0		14	3	
MIB1 index	*n* = 41		0.147	*n* = 41		0.712	*n* = 51		**0.046**	*n* = 71		0.525
<30	10	3		10	3		11	6		24	5	
>=30	26	2		20	8		30	4		37	5	
p53	*n* = 43		0.711	*n* = 43		0.107	*n* = 53		0.456	*n* = 73		0.259
0	29	4		22	11		31	8		50	8	
1+	5	1		6	0		7	3		9	1	
2+	4	0		4	0		4	0		3	2	
Lymph node metastasis	*n* = 41		0.25	*n* = 41		0.238	*n* = 52		0.381	*n* = 69		0.882
Negative	17	1		12	6		21	4		31	5	
Positive	19	4		19	4		20	7		28	5	
Recurrence	*n* = 43		0.432	*n* = 43		0.882	*n* = 53		0.143	*n* = 73		**0.040**
Negative	29	3		24	8		29	10		44	11	
Positive	9	2		8	3		13	1		18	0	

Expression groups derived from semi-proportional score described in Fig.4A. *P* value, Chi-squared test. DCIS, ductal carcinoma in situ; pap, papillotubular carcinoma; sol, solid-tubular carcinoma; sci, scirrhous carcinoma; ER, estrogen receptor; PgR, progesterone receptor LA, luminal A (; ER positive and/or PgR positive, HER2 negative); LB, luminal B (ER positive and/or PgR positive, HER2 positive); TN, triple negative.

Bold indicates values that are statistically significant.

In contrast to lamin A/C or nesprin-2, SUN1 and SUN2 expression status was not associated with any specific pathological classification. However, Table2 shows that loss of SUN1 was observed with a very high frequency in all clinicopathological classifications, suggesting a fundamental role for the loss of SUN1 expression in breast cancer initiation and/or progression.

### *SUN1* and *SUN2* mRNA expression in breast cancer tissue and cultured cells

**Figure 5:**
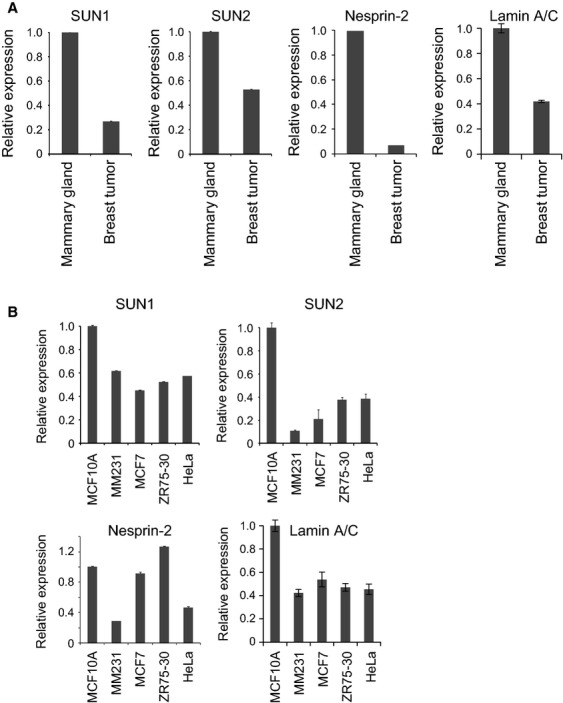
mRNA expression of linker of nucleoskeleton and cytoskeleton (LINC) complex and nuclear lamina components was also reduced in cancer. (A) *SUN1, SUN2, nesprin-2,* and *lamin A/C* mRNA expression in human mammary epithelial and breast tumor tissues was measured by real-time PCR. Results are presented as means ± SD. (B) *SUN1, SUN2, nesprin-2*, and lamin A/CmRNA levels in normal mammary epithelial cells (MCF10A), human breast tumor cells (MDA-MB-231 [described as MM231], MCF7; and ZR75-30), and human cervical cancer cells (HeLa) were analyzed by real-time PCR. The values were normalized against *GAPDH* mRNA. Results are presented as means ± SD.

Next we used real-time PCR to investigate the mRNA expression of LINC complex and nuclear lamina components in human breast tumor. We compared the mRNA expression of these components in RNAs from commercially available human breast tumor and human mammary gland tissue (Fig.5A). The results showed that expression of *SUN1*, *SUN2*, *nesprin-2,* and *lamin A/C* mRNA in breast tumor was lower than mammary gland tissue. Moreover, cDNA from several human breast tumor cell lines contained less *SUN1*, *SUN2*, *nesprin-2* mRNA, and lamin A/C than cDNA from MCF10A, an immortalized but nontumorigenic mammary epithelial cell line widely used as a normal control; a notable exception to this pattern was *nesprin-2* expression in ZR75-30 cells (Fig.5B).

## Discussion

### Reduced expression of a nuclear lamina and LINC complex components in breast cancer tissue

In this study we performed immunohistochemistry to demonstrate reduced expression of four LINC complex and nuclear lamina components, SUN1, SUN2, nesprin-2, and lamin A/C, in breast cancer tissues. Quantitative analysis using immunohistochemistry revealed that expression of all four components tested was significantly lower in tumor regions than in cancer-associated noncancerous regions. To the best of our knowledge, this is the first report showing the loss of SUN1, SUN2, and nesprin-2 in tumor tissues, although the reduced lamin A/C expression in breast cancer tissue is consistent with previous data 12,13. In addition, we also showed that loss of nesprin-2 and lamin A/C protein expression was associated with specific clinicopathological characteristics, suggesting that loss of LINC complex proteins plays a pathological role in breast cancer progression.

### Possible pathological functions of reduced a nuclear lamina protein and LINC complex components expression

The LINC complex and nuclear lamina components play diverse functions such as nuclear structure, nuclear positioning, cell migration, cell cycle, and genome integrity 3,4. The precise functions of reduced LINC complex and nuclear lamina protein expression remains to be elucidated; however, it is possible to envisage several possible pathological functions of the loss of LINC complex and nuclear lamina components in tumor progression. First, robustly reduced expression of these four components might alter the cellular and nuclear structure, thereby contributing to cancer progression. Because the LINC complex and nuclear lamina function in nuclear organization 26 and nuclear mechanical stiffness 27,28, loss of LINC complex and nuclear lamina proteins reduces nuclear and cellular rigidity, and consequently increases tissue fluidity which is crucial for invasive activity. In addition, altered nuclear organization obviously affects genome integrity. To date, nuclear structural defects in cancer cells have been explained by the loss of lamin A/C 12,13. Here, we showed that the loss of lamin A/C in breast cancer tissue was accompanied by the loss of LINC complex components. Importantly, all patients demonstrated decreased expression levels of at least one of four components. These results suggest that the other components, that is, SUN1, SUN2, and nesprin-2, also influence nuclear structure, which controls diverse nuclear processing including genome integrity, proliferation, and cell migration. Second, reduced LINC complex and nuclear lamina protein expression can modify cancer-associated gene expression. Wazir et al. showed that higher lamin A/C mRNA expression in breast cancer tumors was associated with early clinical stages, better clinical outcomes, and better overall and disease-free survival 13. Our results in particular indicated that reduced expression of lamin A/C protein was associated with frequency of recurrence (*P *< 0.05) and HER2 expression (*P *< 0.05); the latter is closely related to breast cancer malignancy. Importantly, both our study and that of Wazir et al. demonstrated that high lamin A/C expression correlates with better clinical outcome. At the moment, the detailed molecular mechanisms underlying these associations are unknown; however, because hundred of genes interact with the nuclear lamina, and lamin A/C regulates gene expression 29,30, it is possible that loss of this protein may alter expression of cancer-related genes, including HER2. In addition, LINC complex components either directly (SUN1 and SUN2) or indirectly (nesprin-2) interact with lamin A/C, thereby potentially controlling lamin A/C function. Third, loss of LINC complex and nuclear lamina components may regulate cell proliferation and promote cancer progression; consistent with this, nesprin-1 and nesprin-2 play roles in cell proliferation 31–33. Our results revealed that loss of nesprin-2 in breast cancer tissue was associated with the MIB1 index (*P *< 0.05), which correlates with cell growth. Fourth and most importantly, because SUN and the nesprin proteins play prominent roles in the DNA damage response, loss of LINC complex components may suppress repair of DNA damage 34–36. Impairment of DNA repair is a crucial event in malignant transformation. We demonstrated significantly reduced expression of SUN1, SUN2, and nesprin-2 proteins in breast cancer (SUN1, 88%; SUN2, 74%; nesprin-2, 79%) regardless of clinicopathological classification (Table2) and all patients exhibited reduced expression of at least one of four components. Therefor the loss of LINC complex or nuclear lamina protein may induce impairment of DNA repair and then play a role in tumor initiation.

### The molecular mechanism underlying robust loss of a nuclear lamina protein and LINC complex proteins

Although some patients exhibited reduced expression of SUN1, but not SUN2 or lamin A/C (Fig.5F), others exhibited the opposite pattern, suggesting that reductions in the expression of each LINC complex component are independent of each other. SUN1 expression is regulated at the level of protein stability 37. An in vitro experiment showed that overexpression or siRNA knock down of each protein did not affect the expression of other LINC complex proteins (data not shown), suggesting that the stabilities of the LINC complex components are independently regulated. Inactivation of the lamin A/C gene by hypermethylation of CpG islands in the promoter has been reported in leukemias and lymphomas 10. Therefore, transcriptional suppression via epigenetic regulation may occur in a gene-specific manner, as shown in lamin A/C expression.

Future studies are required to understand of the detailed pathological functions of loss of the LINC complex and nuclear lamina components and deformation of nuclear structure during malignant transformation. Such knowledge will, in turn, provide the basis for developing new diagnostic tools and therapeutics. Moreover, although the loss of lamin A/C has been proposed as a novel diagnostic marker in several cancers, our results suggest the possibility of using a combination of LINC complex-related components for both prognostic and diagnostic purposes.

## Supporting Information

**Figure S1.** Validation of antibodies.

**Figure S2.** Kaplan–Meier analysis of LINC complex component expression levels in breast cancer patients.

**Table S1.** Cinicopathological parameters (DCIS/micro invasives were excluded).
