# Supplementary material for: Global loss of a nuclear lamina component, lamin A/C, and LINC complex components SUN1, SUN2, and nesprin-2 in breast cancer
Source: Cancer Med. 2015 Jul 14;4(10):1547–57. doi: 10.1002/cam4.495 (PMC4618625; doi:10.1002/cam4.495)

Matsumoto et al., Supplemental Figure.2
Validation of anti-SUN1, anti-SUN2, and anti-nesprin-2 antibodies.

A

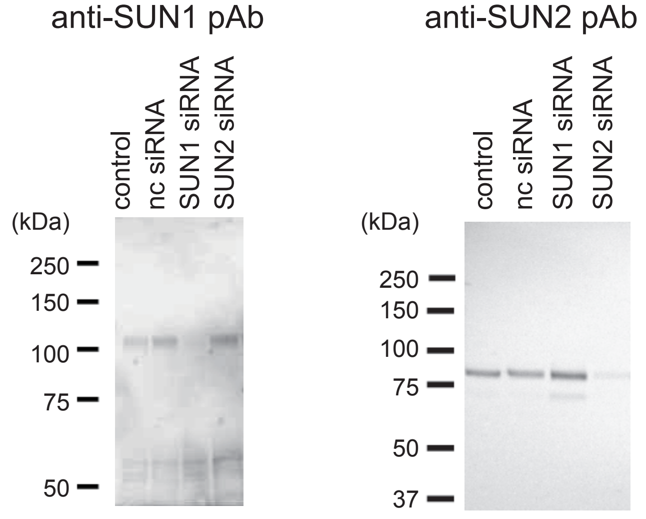

B

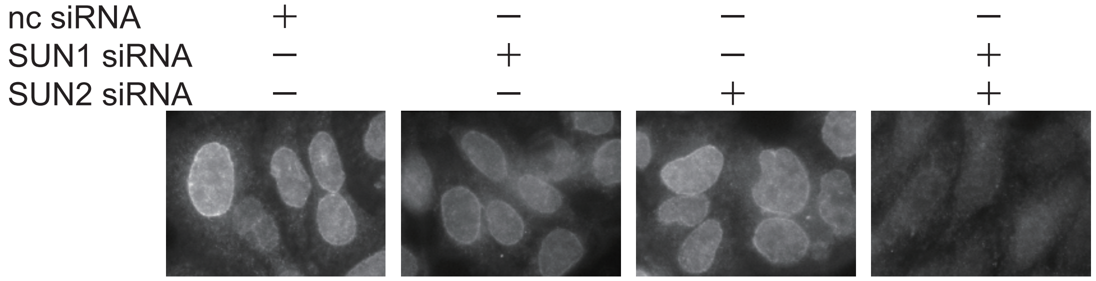

Supplement: Supplementary file 1 [file cam40004-1547-sd1.pdf]
