# Supplementary figures and images for: Global loss of a nuclear lamina component, lamin A/C, and LINC complex components SUN1, SUN2, and nesprin-2 in breast cancer
Source: Cancer Med. 2015 Jul 14;4(10):1547–57. doi: 10.1002/cam4.495 (PMC4618625; doi:10.1002/cam4.495)

Kaplan-Meier analysis of LINC complex component expression levels in breast cancer patients

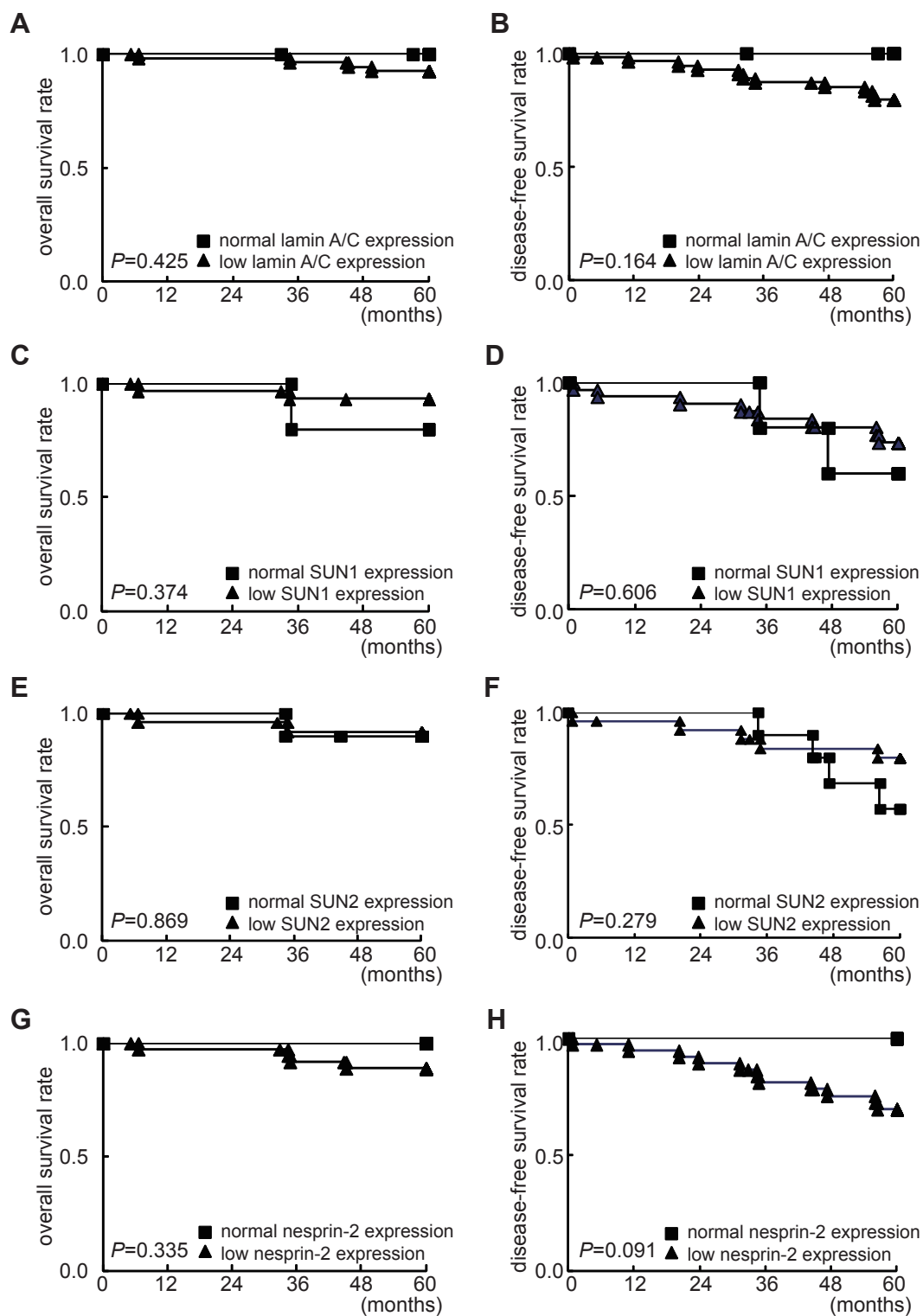

Supplement: Supplementary file 2 [file cam40004-1547-sd2.pdf]
