# Supplementary material for: Global loss of a nuclear lamina component, lamin A/C, and LINC complex components SUN1, SUN2, and nesprin-2 in breast cancer
Source: Cancer Med. 2015 Jul 14;4(10):1547–57. doi: 10.1002/cam4.495 (PMC4618625; doi:10.1002/cam4.495)
Supplement: Supplementary file 3 [file cam40004-1547-sd3.pdf]

Table S1 Cinicopathological parameters (DCIS/micro invasives were excluded)

	SUN1			SUN2			nesprin-2			lamin A/C		
**group**	low	normal		low	normal		low	normal		low	normal	
	n=34	n=5		n=28	n=11		n=39	n=7		n=53	n=10	
	87.2%	12.8%	*P*	71.8%	28.2%	*P*	84.8%	15.2%	*P*	84.1%	15.9%	*P*
**Nuclear grade**	n=38		0.089	n=38		0.95	n=45		0.860	n=61		0.734
Grades 1/2	20	1		15	6		21	3		32	5	
Grade 3	13	4		12	5		18	3		20	4	
**Alive/Dead**	n=36		0.670	n=36		0.511	n=44		0.295	n=58		0.268
Alive	27	4		23	8		32	6		43	9	
Dead	4	1		3	2		6	0		6	0	
**ER**	n=38		0.819	n=38		0.508	n=45		0.114	n=61		0.854
negative	15	2		13	4		20	1		22	4	
positive	18	3		14	7		19	5		29	6	
**PgR**	n=37		0.677	n=37		0.641	n=44		0.905	n=59		0.676
negative	16	2		12	6		20	3		24	5	
positive	16	3		14	5		18	3		26	4	
**HER2**	n=39		0.976	n=39		0.821	n=46		**0.042***	n=62		0.051
0/1+	27	4		22	9		31	3		38	10	
2+/3+	7	1		6	2		8	4		14	0	
**Intrinsic subtype**	n=37		0.659	n=37		0.926	n=44		**0.028***	n=60		0.317
LA	14	3		11	6		16	2		24	6	
LB	4	0		3	1		3	3		8	0	
HER2-like	3	1		3	1		5	1		7	0	
TN	11	1		9	3		14	0		12	3	
**MIB1 index**	n=37		0.074	n=37		0.983	n=44		0.720	n=60		0.599
<30	7	3		7	3		10	2		18	4	
≧30	25	2		19	8		28	4		33	5	
**p53**	n=39		0.662	n=39		0.100	n=46		0.589	n=62		0.308
0	26	4		19	11		28	7		41	7	
1+	4	1		5	0		7	2		8	1	
2+	4	0		4	0		4	0		3	2	
**Lymph node metastasis**	n=37		0.314	n=37		0.142	n=45		0.081	n=58		0.890
negative	14	1		9	6		19	1		23	4	
positive	18	4		18	4		19	6		26	5	
**Recurrence**	n=39		0.431	n=39		0.884	n=46		0.088	n=62		**0.042***
negative	26	3		21	8		27	7		36	10	
positive	8	2		7	3		12	0		16	0	

Expression groups derived from semi-proportional score described in Fig. 2B. *P* value, Chi-squared test
DCIS, ductal carcinoma in situ;
idc, invasive ductal carcinoma;
pap, papillotubular carcinoma; sol, solid-tubular carcinoma; sci, scirrhous carcinoma;
ER, estrogen receptor; PgR, progesterone receptor;
luminal, ER positive and/or PgR positive.
Bold indicates values that are statistically significant.
